# Biomechanism of chlorogenic acid complex mediated plasma free fatty acid metabolism in rat liver

**DOI:** 10.1186/s12906-016-1258-y

**Published:** 2016-08-05

**Authors:** Sudeep H.V., Venkatakrishna K, Dipak Patel, Shyamprasad K

**Affiliations:** Vidya Herbs (P) Ltd., R&D Center for Excellence Jigani Industrial Area, Anekal Taluk, Bangalore, Karnataka India

**Keywords:** Lipid metabolism, Triglycerides, Liver, Chlorogenic acid, AMPK

## Abstract

**Background:**

Plasma free fatty acids (FFA) are involved in blood lipid metabolism as well as many health complications. The present study was conducted to evaluate the potential role of chlorogenic acid complex from green coffee bean (CGA7) on FFA metabolism in high fat diet fed rats.

**Methods:**

Hyperlipidemia was induced in Wistar rats using high-fat diet. The animals were given CGA7/orlistat concurrently for 42 days. The parameters analysed during the study include plasma and liver total cholesterol (TC), Triglycerides (TG) and FFA. AMPK activation in the liver was analysed through ELISA. The multiple factors involved in AMPK mediated FFA metabolism were analysed using western blotting.

**Results:**

CGA7 (50, 100, 150 mg/kg BW) decreased triglycerides (TG) and FFA levels in plasma and liver. CGA7 administration led to the activation of AMP-activated protein kinase (AMPK) and a subsequent increase in the levels of carnitine palmitoyltransferase 1 (CPT-1). There was a decrease in acetyl-CoA carboxylase (ACC) activity as evident by the increase in its phosphorylation level.

**Conclusion:**

Chlorogenic acids improved the blood lipid metabolism in rats by alleviating the levels of FFA and TG, modulating the multiple factors in liver through AMPK pathway. The study concludes that CGA7 complex can be promoted as an active ingredient in nutrition for obesity management.

**Electronic supplementary material:**

The online version of this article (doi:10.1186/s12906-016-1258-y) contains supplementary material, which is available to authorized users.

## Background

Fatty acids in the plasma (FFA) play a crucial role in metabolic syndrome such as obesity and type 2 diabetes [1, 2]. Plasma FFA levels are usually elevated in obesity due to the fact that enlarged adipose tissue mass releases more FFA and its clearance reduced. Furthermore, the anti-lipolytic action of insulin is inhibited by plasma FFA, which also increases the rate of FFA release into the circulation [2]. Glucose uptake via insulin signalling is affected by FFA [3]. Oxidation of FFA to form ATP, or esterification for storage as triglycerides (TG) occurs in the liver [4]. Fatty liver is a consequence of elevated plasma FFA resulting in intracellular accumulation of lipid metabolites. Therefore, reducing the level of plasma FFA and promoting FFA uptake and oxidation in the liver would be a potential strategy in the management of metabolic syndrome.

Chlorogenic acids (CGA) represent a large family of phenolic compounds formed by the esterification of cinnamic acids with quinic acids. Green coffee is a major source of CGA in nature [5]. Green coffee beans have received much attention in the west mainly due to the beneficial effects of CGA against obesity and metabolic disorders such as type 2 diabetes. A large body of evidence suggest that CGA exhibits anti-obesity activity by alleviating lipid metabolism in high-fat diet-fed rats [6]. Recent studies showed that CGA stimulated glucose transport via increasing expression of GLUT4 and PPAR-γ transcript. This is due to the activation of AMP activated protein kinase (AMPK). CGA regulates glucose and lipid metabolism and improves insulin sensitivity. However, there are no scientific reports explaining the detailed mechanism of action of CGA complex on FFA metabolism.

The present study investigates the hypolipidemic effects of CGA7, a complex of seven isomers of chlorogenic acid from green coffee beans supplied by Department of Phytochemistry, Vidya Herbs Pvt Ltd., in rats.

## Methods

### Reagents

All the reagents and chemicals were purchased from Sigma Aldrich Ltd. (India) unless otherwise stated. Antibodies for AMP-activated protein kinase α (AMPKα) and its phosphorylated form (pAMPKα), acetyl CoA carboxylase (ACC) and phosphor-ACC (pACC), carnitine palmitoyltransferase 1 (CPT-1), and β-actin were purchased from Santa Cruz Biotechnology (Santa Cruz, CA).

### Experimental design

Forty-eight male, four week old Sprague-Dawley rats (Biogen, Bangalore, India) were housed in stainless-steel cages and placed in a room under a controlled atmosphere (temperature, 22 ± 1 °C, humidity, 55 ± 5 %; 12 h light/dark cycle). During a 1-week acclimatization period, all rats consumed a commercial diet and tap water *ad libitum*. The animals were divided into six groups (*n* = 6): except control animals, all other groups were given in-house prepared high-fat-diet with or without orlistat or CGA7 (50, 100 and 150 mg/kg b.w.) (Table 1). CGA7 and orlistat were dissolved in water for oral administration to rats. The diets were given in the form of pellets for six weeks. The animal studies were carried out after the CPCSEA (Committee for the purpose of control and supervision of experiments on animals, a statutory committee established under the Prevention of Cruelty to Animals Act, 1960 in India) approval through Institutional Animal Ethical Committee (VHPL/PCL/IAEC/02/13) independently formed by CPCSEA.

**Table 1:** Experimental design for the study

Groups	Treatment and dose (mg/kg b.w)
I	Control (Normal pellet diet)
II	High fat diet
III	High fat diet + Orlistat (30 mg/kg)
IV	High fat diet + CGA7 (50 mg/kg)
V	High fat diet + CGA7 (100 mg/kg)
VI	High fat diet + CGA7 (150 mg/kg)

### Determination of total cholesterol (TC), TG and FFA in plasma and liver tissue

A 10 % w/v liver homogenate was prepared using 0.15 M KCl, centrifuged at 1000 g for 10 min at 4 °C. The supernatant was for biochemical analysis. Plasma and liver TC and TG were measured enzymatically using commercial kit (ROBONIK Prietest kit) in autoanalyzer (Unitron Biomed). FFA was determined by GC-MS (Schimadzu QP2010).

### Determination of AMPK phosphorylation using ELISA

The activation of AMPK in liver homogenates was determined using AMPK (pT172) ELISA kit from Life technologies. The experiment was conducted as per manufacturers’ instructions. The total assay incubation time was only 4 h. Tetramethyl benzidine (TMB) was used as substrate solution. The optical density was read on a standard microplate reader (Multiskan EX Thermoscientific).

### Western blot analysis

Proteins from the liver tissues were extracted with a RIPA buffer (50 mM Tris-HCl, pH 7.4, 150 mM NaCl, 1 % NP-40, 0.25 % deoxycholic acid, 1 mM EDTA) supplemented with 1 mM phenylmethylsulfonyl fluoride. The concentration of protein in the liver homogenates was determined by the Bradford method. The proteins were electrophoresed on SDS-PAGE, and transferred onto polyvinylidene difluoride (PVDF) membranes. The membranes were blocked in 2 % BSA in TBS-T (50 mmol/L Tris HCl, pH 7.5, 150 mmol/L NaCl, 0.1 % Tween 20) for 1 h at room temperature. The membranes were kept overnight at 4 °C with primary antibodies followed by washing thrice with TBS-T (6 min each). The secondary antibody incubation was for 1 h at room temperature. After applying the substrate, the blots were developed using X-ray films. Beta actin was used as a loading control.

### Statistical analysis

The data were expressed as mean ± SEM and analyzed by one way ANOVA followed by Dunnett’s t test using Graphpad Prism version 5. The data were considered statistically significant at *p* < 0.05.

## Results and discussion

### CGA7 promoted weight loss and improved the lipid profile in HFD-fed rats

**Fig. 1:**
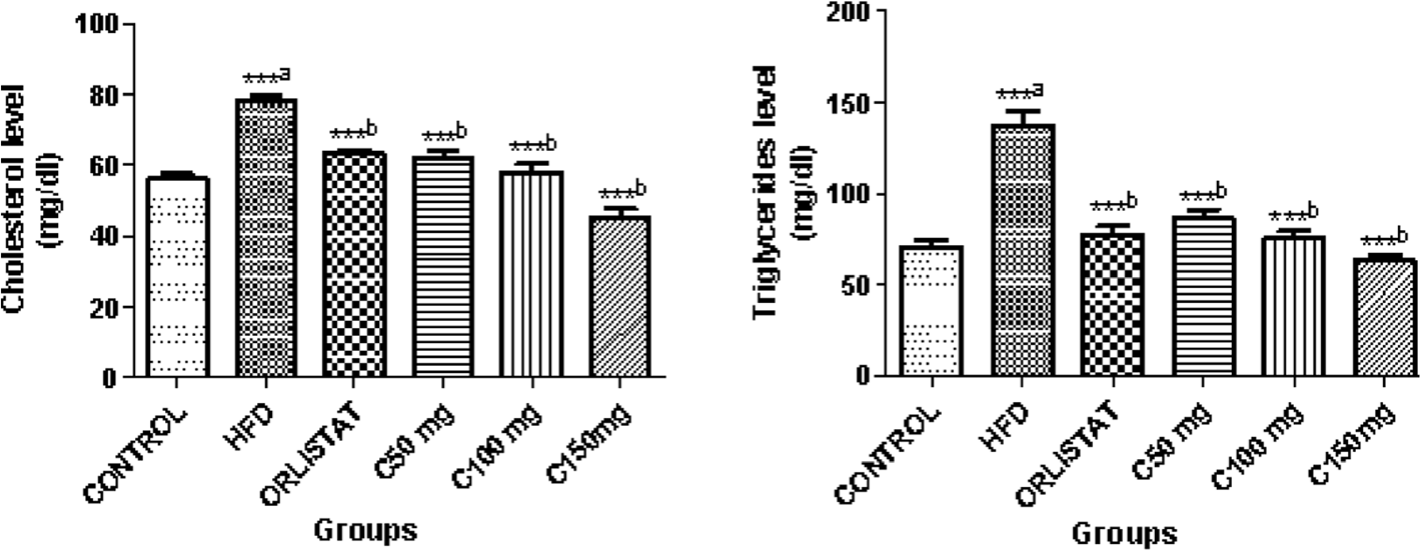
Effect of CGA7 on plasma total cholesterol and triglyceride. Data were analyzed by one way ANOVA followed by Dunnett’s *t* test. Number of animals in each group *n* = 6. ^a^Comparison made with control group. ^b^Comparison made with high fat diet group. ***P < 0.001, **P < 0.01, *P < 0.05

**Fig. 2:**
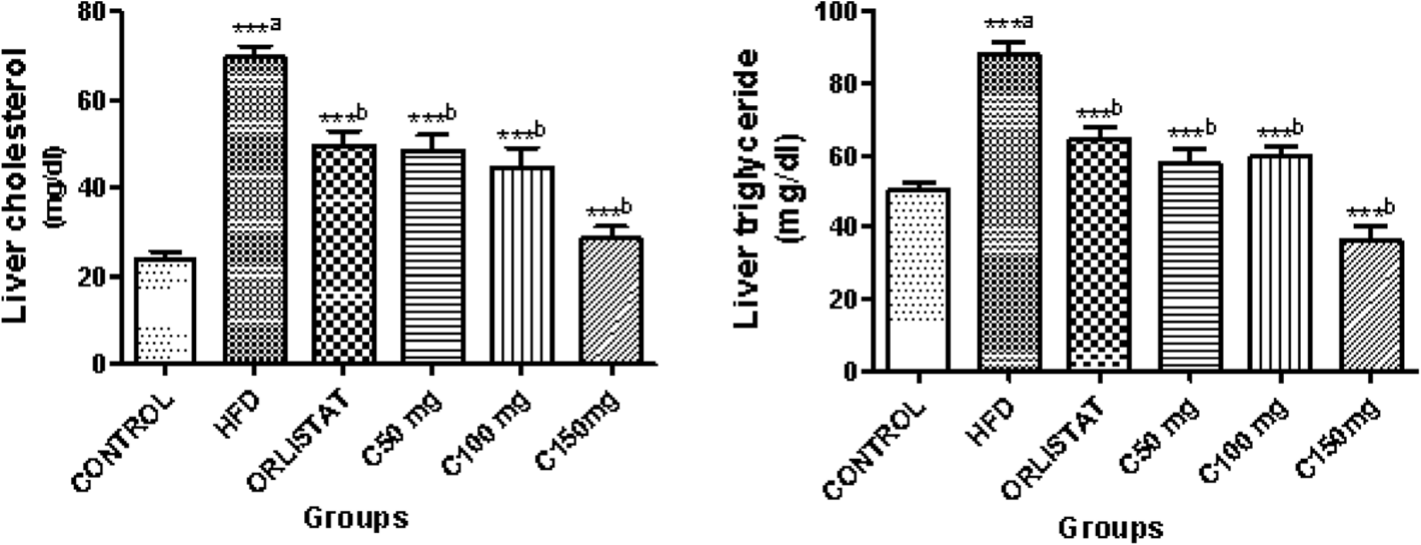
Effect of CGA 7 on liver cholesterol & triglycerides. Data were analyzed by one way ANOVA followed by Dunnett’s *t* test. Number of animals in each group *n* = 6. ^a^Comparison made with control group. ^b^Comparison made with high fat diet group. ***P < 0.001

**Fig. 3:**
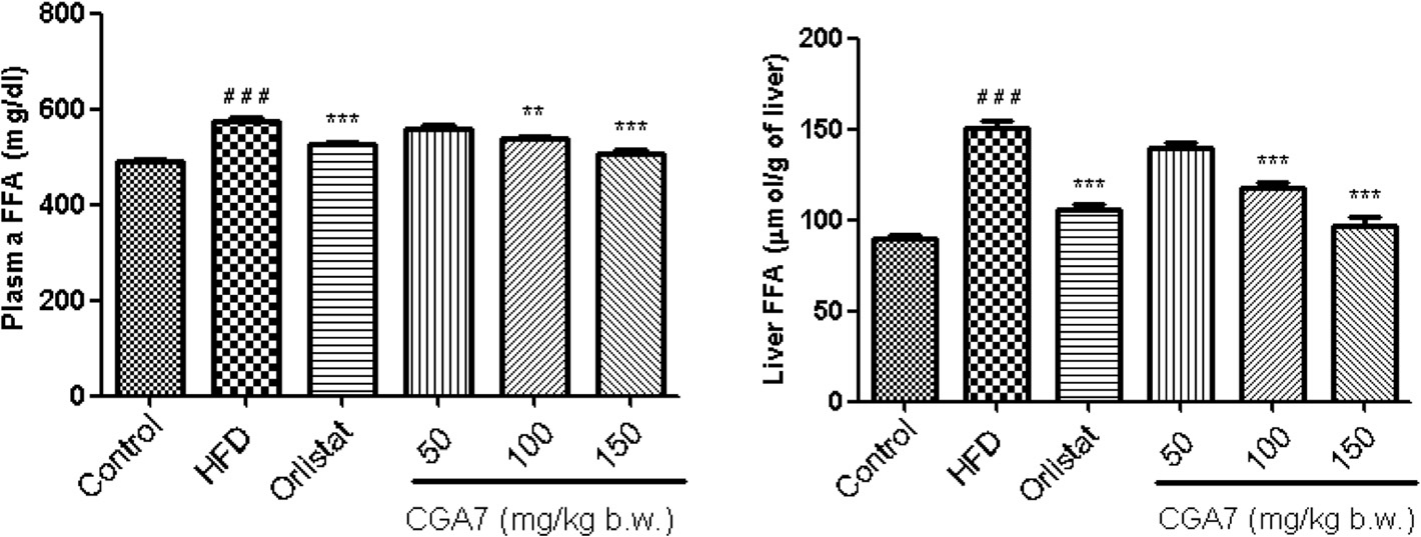
Effects of CGA7 complex on plasma and liver FFA levels in hyperlipidemic rats. Data were analyzed by one way ANOVA followed by Dunnett’s *t* test. Number of animals in each group *n* = 6. #, *p* < 0.05 compared with control group. *p < 0.05 compared with HFD control

The high fat diet fed rats had gained considerably more body weight than the normal rats. CGA7 treatment however restored the body weight to normal levels in HFD rats (Table 2). After 42 days of treatment, the HFD-fed rats showed elevated levels of liver and plasma cholesterol, triglycerides and free fatty acids (*p* < 0.001) as compared to the normal rats (Figs. 1 & 2). There was considerable improvement in the lipid metabolism observed in treatment groups including Orlistat and CGA7 supplementation (*p* < 0.001) (Figs. 1 & 3). Furthermore, CGA7 administration decreased the liver weight and epididymal fat in HFD-fed rats (Table 2).

**Table 2:** Influence of CGA7 complex on weight gain and metabolic variables in HFD- fed rats

Parameter	Control	High fat diet	High fat diet + orlistat (30 mg/kg b.w.)	High fat diet + CGA7 (50 mg/kg b.w.)	High fat diet + CGA7 (100 mg/kg b.w.)	High fat diet + CGA7 (150 mg/kg b.w.)
Body weight gain (g)	68.41 ± 1.18	112.0 ± 4.51^# # #^	88.01 ± 3.68***	75.65 ± 3.44***	65.88 ± 2.14***	60.63 ± 2.89***
Food intake (g/day)	14.93 ± 0.77	8.26 ± 0.51^# # #^	8.54 ± 0.40	7.78 ± 0.45	8.96 ± 0.45*	6.98 ± 0.35
Liver weight (g)	3.87 ± 0.18	4.50 ± 0.19^#^	3.47 ± 0.11***	3.56 ± 0.10***	3.57 ± 0.12***	3.39 ± 0.10***
Mesenteric fat (g)	0.59 ± 0.06	1.31 ± 0.12^# # #^	0.98 ± 0.03*	1.01 ± 0.07*	1.0 ± 0.04*	1.00 ± 0.07*
Epididymal fat (g)	0.58 ± 0.02	2.07 ± 0.12^# # #^	1.26 ± 0.05*	1.06 ± 0.03***	0.84 ± 0.02***	0.74 ± 0.02**

Data represent the means ± SEM (*n* = 6). Data were analysed using one way Anova for analysing the differences between groups (^#^, *p* < 0.05, ^# # #^, *p* < 0.001) compared with control group. *, *p* < 0.05, **, *p* < 0.01 and ***, *p* < 0.001 compared with HFD group

### CGA7 modulated the regulatory enzymes of FFA catabolism in liver of HFD rats

**Fig. 4:**
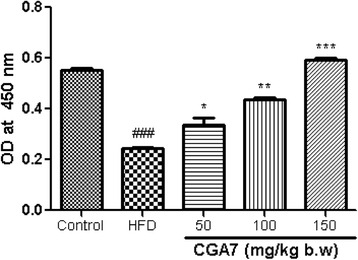
Regulation of AMPK by CGA7 in rat liver. Data are mean ± SEM values of three individual experiments. The values were compared with the control using analysis of variance followed by unpaired student’s *t* tests. ^#^ *p* < 0.05, significant differences from the normal control group. *p < 0.05, significant differences from the positive control group

**Fig. 5:**
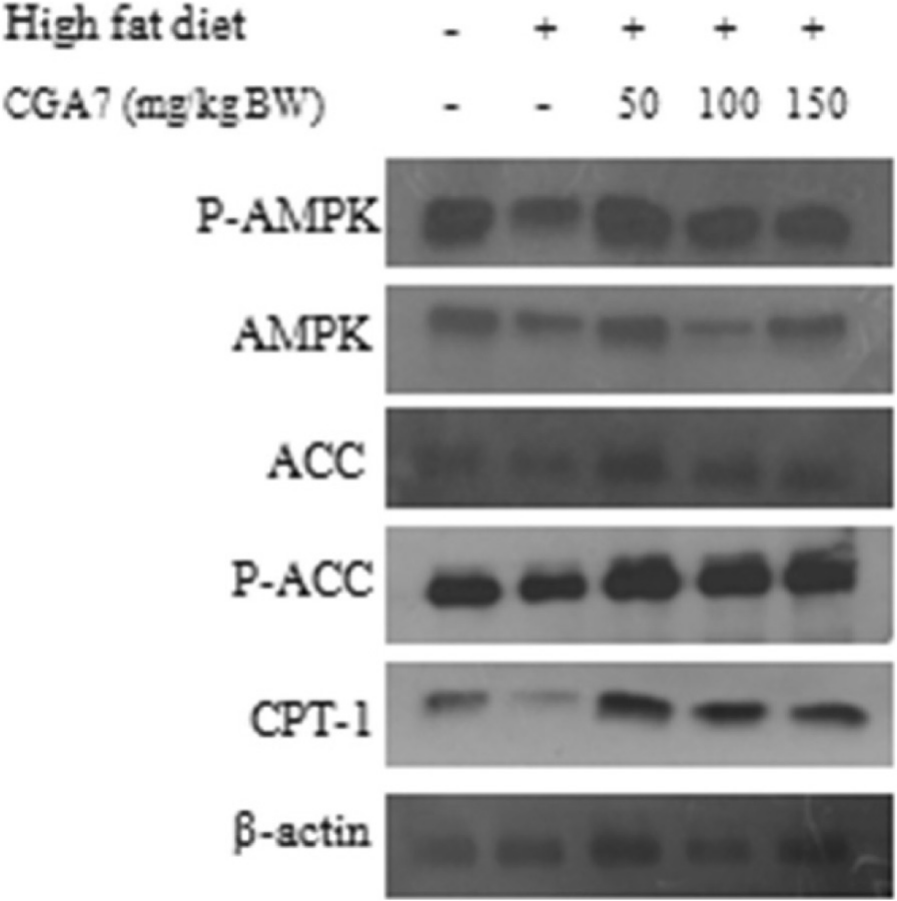
Effects of CGA7 on the proteins expression of AMPK, CPT1 and ACC in liver of HFD fed rats

CGA7 complex significantly increased the phosphorylated form of AMPK in the liver of HFD-fed rats (*P* < 0.01) (Fig. 4) as evident through ELISA. It was further confirmed using western blotting (Fig. 5). CGA7 treatment also influenced the expression of CPT-1 and ACC. CPT-1 plays an important role in FFA β-oxidation. ACC is a vital enzyme required for FFA synthesis, respectively, in liver. CGA7 increased significantly the expression of CPT-1 and the level of ACC phosphorylation (Fig. 5). These results indicate collectively that chlorogenic acids promote the FFA uptake and oxidation, and inhibit FFA accumulation by modulating the vital enzymes expression in liver of hyperlipidemic rats.

The study investigated the effect of CGA7 on free fatty acid metabolism in high-fat diet induced obese rats. There are studies describing the anti-obesity mechanisms of chlorogenic acids (CGA) [6, 7]. Rodriguez de Sotillo and Hadley [8] reported that CGA reduced the TG and TC in blood. However, the mechanism is not yet clear. FFA plays an essential role in regulating triglyceride metabolism [9]. The elevated levels of plasma FFA leads to formation of fatty liver and hyperlipidemia [10]. There are no reports on the effect of CGA on FFA metabolism. In the present study, CGA7 complex was shown to influence the FFA metabolism attributing to the synergistic effect of chlorogenic acid isomers. The presence of seven isomers of chlorogenic acids was confirmed by LCMS (Data not shown). Earlier reports suggest that 5-Caffeoylquinic acid (5-CQA), one of the isomers of CGA, influence the glucose transport in skeletal muscle through the activation of AMPK and down-regulation of ACC [11]. 5-CQA also improves the lipid metabolism through the regulation of AMPK [12]. Our data for the first time demonstrated that CGA7 complex of chlorogenic acids exert anti-hyperlipidemic effect by promoting FFA catabolism.

There was an increase in body weight (Table 2), plasma and liver TC, TG levels in high-fat diet fed rats compared to rats in normal control group. Our study for the first time showed that CGA7 complex decreased plasma FFA levels in hyperlipidemic rats via AMPK activation. Activated form of AMPK plays a pivotal role in intracellular lipid metabolism [13]. In the present study, CGA7 complex treated rats showed an upregulation in the activated AMPK levels compared to high-fat diet fed animals. Further there was an increase in CPT-1 level and a decreased expression of pACC indicating an inhibition of ACC activity. Inhibition of ACC activity is related to a decreased malonyl-CoA which would otherwise inhibits the activity of CPT-1. CPT-1 plays a crucial role in the FFA catabolism through β-oxidation [14]. In order for lipids to be used as fuel, FFA must be converted intracellularly into long-chain fatty-acyl-CoAs through β-oxidation. Decreased ACC activity can be correlated to the suppression of FFA synthesis and the progression of FFA β-oxidation [15].

Interestingly, we found that the efficacy of CGA7 was more pronounced at 50 mg/kg b.w. dosage group than the higher doses of CGA7 (Fig. 5). This is in agreement with previous studies from Ong et al. [11] wherein ACC phosphorylation was more prominent with the lower concentration of CGA. The current findings clearly indicate the efficacy of CGA7 on FFA catabolism via activation of AMPK.

## Conclusion

The effect of isomers of chlorogenic acids in green coffee bean on FFA metabolism and its possible mechanism has been addressed for the first time. CGA promotes FFA catabolism by regulating the AMPK pathway.

## Abbreviations

5-CQA, 5-Caffeoyl quinic acid; ACC, acetyl CoA carboxylase; AMPK, AMP activated protein kinase; CGA, chlorogenic acids; CGA7, chlorogenic acid complex; CPT-1, carnitine palmitoyl transferase-1; FFA, plasma free fatty acids; LCMS, liquid chromatography-mass spectrometry; pACC, phosphorylated acetyl CoA carboxylase; pAMPK, phosphorylated acetyl CoA carboxylase; TC, total cholesterol; TG, triglycerides

## Additional file

Additional file 1: Available data and materials. (DOCX 24 kb)
