# Supplementary material for: Biomechanism of chlorogenic acid complex mediated plasma free fatty acid metabolism in rat liver
Source: BMC Complement Altern Med. 2016 Aug 5;16:274. doi: 10.1186/s12906-016-1258-y (PMC4974694; doi:10.1186/s12906-016-1258-y)
Supplement: Additional file 1: — Available data and materials. (DOCX 24 kb) [file 12906_2016_1258_MOESM1_ESM.docx]

Table 1. Individual animal data for body weight gain (g)

| Control | Hyperlipidemia | Hyperlipidemia  + orlistat  (30 mg/kg b.w.) | Hyperlipidemia  +CGA7  (50 mg/kg b.w.) | Hyperlipidemia  +CGA7  (100 mg/kg b.w.) | Hyperlipidemia  +CGA7  (150 mg/kg b.w.) |
| --- | --- | --- | --- | --- | --- |
| 65.23 | 110.46 | 90.24 | 81.93 | 66.00 | 55.92 |
| 70.25 | 133.53 | 98.07 | 74.92 | 78.75 | 58.68 |
| 68.25 | 97.15 | 97.49 | 91.19 | 64.11 | 58.24 |
| 68.69 | 100.51 | 99.22 | 71.06 | 68.19 | 76.96 |
| 65.36 | 103.07 | 88.45 | 79.37 | 64.98 | 68.34 |
| 71.25 | 110.23 | 82.33 | 80.75 | 61.48 | 51.52 |
| 73.89 | 113.52 | 70.86 | 62.32 | 57.95 | 56.70 |
| 64.35 | 127.41 | 77.44 | 63.69 | 65.57 | 58.69 |

Table 2. Individual animal data for food intake (g/day)

| Control | Hyperlipidemia | Hyperlipidemia  + orlistat  (30 mg/kg b.w.) | Hyperlipidemia  +CGA7  (50 mg/kg b.w.) | Hyperlipidemia  +CGA7  (100 mg/kg b.w.) | Hyperlipidemia  +CGA7  (150 mg/kg b.w.) |
| --- | --- | --- | --- | --- | --- |
| 13.41 | 5.93 | 7.91 | 6.42 | 7.79 | 8.06 |
| 12.46 | 6.91 | 3.09 | 2.98 | 8.96 | 6.73 |
| 9.80 | 2.92 | 9.43 | 7.40 | 8.50 | 6.12 |
| 11.70 | 5.96 | 8.89 | 6.90 | 7.54 | 6.55 |
| 12.70 | 9.92 | 8.69 | 5.58 | 8.30 | 9.37 |
| 12.97 | 7.91 | 8.30 | 9.29 | 9.20 | 9.49 |
| 10.56 | 4.85 | 8.33 | 5.86 | 8.20 | 6.34 |
| 18.39 | 10.75 | 12.31 | 12.43 | 7.65 | 6.17 |

Table 3. Individual animal data for liver weight (g)

| Control | Hyperlipidemia | Hyperlipidemia  + orlistat  (30 mg/kg b.w.) | Hyperlipidemia  +CGA7  (50 mg/kg b.w.) | Hyperlipidemia  +CGA7  (100 mg/kg b.w.) | Hyperlipidemia  +CGA7  (150 mg/kg b.w.) |
| --- | --- | --- | --- | --- | --- |
| 3.56 | 4.72 | 3.29 | 3.60 | 4.12 | 3.15 |
| 4.28 | 5.40 | 3.61 | 3.63 | 3.43 | 3.20 |
| 4.07 | 4.30 | 3.45 | 3.86 | 3.35 | 3.76 |
| 3.73 | 4.81 | 4.21 | 3.92 | 3.18 | 2.98 |
| 3.83 | 4.00 | 3.27 | 3.24 | 3.77 | 3.79 |
| 3.11 | 4.84 | 3.30 | 3.06 | 3.25 | 3.38 |
| 3.64 | 4.13 | 3.41 | 3.45 | 3.95 | 3.46 |
| 4.77 | 3.77 | 3.18 | 3.69 | 3.51 | 3.40 |

Table 4. Individual animal data for mesenteric fat (g)

| Control | Hyperlipidemia | Hyperlipidemia  + orlistat  (30 mg/kg b.w.) | Hyperlipidemia  +CGA7  (50 mg/kg b.w.) | Hyperlipidemia  +CGA7  (100 mg/kg b.w.) | Hyperlipidemia  +CGA7  (150 mg/kg b.w.) |
| --- | --- | --- | --- | --- | --- |
| 0.44 | 0.92 | 1.06 | 1.10 | 1.20 | 0.96 |
| 0.46 | 1.50 | 0.99 | 0.93 | 0.95 | 1.15 |
| 0.76 | 0.97 | 1.16 | 1.17 | 0.95 | 0.59 |
| 0.47 | 1.50 | 0.85 | 1.09 | 1.13 | 0.97 |
| 0.62 | 1.02 | 1.02 | 0.89 | 0.83 | 0.89 |
| 0.57 | 1.23 | 0.96 | 0.85 | 0.97 | 1.27 |
| 0.56 | 1.35 | 0.81 | 0.68 | 0.85 | 0.96 |
| 0.88 | 1.98 | 0.95 | 1.31 | 1.11 | 1.16 |

Table 5. Individual animal data for epididymal fat (g)

| Control | Hyperlipidemia | Hyperlipidemia  + orlistat  (30 mg/kg b.w.) | Hyperlipidemia  +CGA7  (50 mg/kg b.w.) | Hyperlipidemia  +CGA7  (100 mg/kg b.w.) | Hyperlipidemia  +CGA7  (150 mg/kg b.w.) |
| --- | --- | --- | --- | --- | --- |
| 0.65 | 2.01 | 1.29 | 1.06 | 0.84 | 0.68 |
| 0.53 | 2.13 | 1.1 | 1.01 | 0.75 | 0.73 |
| 0.51 | 1.98 | 1.31 | 1.12 | 1.03 | 0.74 |
| 0.65 | 1.94 | 1.4 | 1.21 | 0.85 | 0.67 |
| 0.43 | 1.8 | 1.25 | 0.96 | 0.77 | 0.87 |
| 0.61 | 2.3 | 1.27 | 0.98 | 0.86 | 0.79 |
| 0.53 | 2.4 | 1.29 | 1.06 | 0.84 | 0.7 |
| 0.76 | 1.96 | 1.2 | 1.04 | 0.87 | 0.74 |

Table 6. ELISA data for AMPK activation in liver homogenates

| Control | Hyperlipidemia | Hyperlipidemia  +CGA7  (50 mg/kg b.w.) | Hyperlipidemia  +CGA7  (100 mg/kg b.w.) | Hyperlipidemia  +CGA7  (150 mg/kg b.w.) |
| --- | --- | --- | --- | --- |
| 0.562 | 0.238 | 0.312 | 0.428 | 0.582 |
| 0.543 | 0.250 | 0.364 | 0.443 | 0.601 |
| 0.55 | 0.315 | 0.374 | 0.53 | 0.619 |
| 0.548 | 0.229 | 0.402 | 0.456 | 0.542 |

Table 7. Individual animal data for plasma FFA

| Control | Hyperlipidemia | Hyperlipidemia  + orlistat  (30 mg/kg b.w.) | Hyperlipidemia  +CGA7  (50 mg/kg b.w.) | Hyperlipidemia  +CGA7  (100 mg/kg b.w.) | Hyperlipidemia  +CGA7  (150 mg/kg b.w.) |
| --- | --- | --- | --- | --- | --- |
| 484.5 | 587 | 535 | 550 | 527 | 501 |
| 489.6 | 590 | 543 | 564 | 538 | 497 |
| 480 | 589 | 512 | 574 | 542 | 513 |
| 475 | 592 | 521 | 560 | 553 | 543 |
| 510 | 590 | 505 | 551 | 517 | 516 |
| 502 | 520 | 550 | 552 | 530 | 488 |
| 488 | 548 | 508 | 542 | 560 | 495 |
| 485 | 585 | 530 | 588 | 532 | 506 |

Table 8. Individual animal data for liver FFA

| Control | Hyperlipidemia | Hyperlipidemia  + orlistat  (30 mg/kg b.w.) | Hyperlipidemia  +CGA7  (50 mg/kg b.w.) | Hyperlipidemia  +CGA7  (100 mg/kg b.w.) | Hyperlipidemia  +CGA7  (150 mg/kg b.w.) |
| --- | --- | --- | --- | --- | --- |
| 84.8 | 153.5 | 104.0 | 139.0 | 118.0 | 94.0 |
| 86.0 | 165.0 | 120.0 | 145.0 | 105.0 | 104.0 |
| 78.0 | 141.0 | 98.0 | 150.0 | 135.0 | 88.0 |
| 95.0 | 140.0 | 102.0 | 126.0 | 113.0 | 80.6 |
| 101.0 | 135.0 | 110.0 | 129.0 | 108.0 | 90.0 |
| 88.0 | 150.0 | 108.0 | 135.0 | 115.0 | 85.0 |
| 91.0 | 163.0 | 105.0 | 139.0 | 123.0 | 120.0 |
| 92.0 | 158.0 | 99.6 | 152.0 | 121.0 | 112.0 |

Table 9. Individual animal data for plasma cholesterol

| Control | Hyperlipidemia | Hyperlipidemia  + orlistat  (30 mg/kg b.w.) | Hyperlipidemia  +CGA7  (50 mg/kg b.w.) | Hyperlipidemia  +CGA7  (100 mg/kg b.w.) | Hyperlipidemia  +CGA7  (150 mg/kg b.w.) |
| --- | --- | --- | --- | --- | --- |
| 60.13 | 78.53 | 63.93 | 65.88 | 63.49 | 50.27 |
| 50.45 | 85.12 | 58.64 | 67.72 | 59.02 | 53.35 |
| 51.75 | 76.87 | 67.36 | 66.23 | 65.14 | 53.60 |
| 54.40 | 78.98 | 61.25 | 61.43 | 52.48 | 35.89 |
| 60.65 | 80.50 | 62.32 | 53.59 | 50.89 | 41.63 |
| 60.07 | 71.21 | 63.76 | 65.24 | 52.01 | 39.33 |
| 53.09 | 76.13 | 63.82 | 60.19 | 66.07 | 41.02 |
| 57.47 | 77.32 | 65.95 | 57.59 | 56.20 | 46.49 |

Table 10. Individual animal data for plasma triglycerides

| Control | Hyperlipidemia | Hyperlipidemia  + orlistat  (30 mg/kg b.w.) | Hyperlipidemia  +CGA7  (50 mg/kg b.w.) | Hyperlipidemia  +CGA7  (100 mg/kg b.w.) | Hyperlipidemia  +CGA7  (150 mg/kg b.w.) |
| --- | --- | --- | --- | --- | --- |
| 75.94 | 100.26 | 75.37 | 109.14 | 86.03 | 60.49 |
| 71.59 | 157.22 | 63.18 | 95.76 | 58.54 | 69.56 |
| 82.38 | 130.77 | 73.06 | 84.62 | 77.27 | 53.99 |
| 85.41 | 158.05 | 60.32 | 89.30 | 62.15 | 61.97 |
| 60.65 | 113.58 | 84.50 | 97.15 | 82.10 | 78.73 |
| 68.58 | 145.77 | 80.39 | 68.86 | 65.79 | 60.95 |
| 61.53 | 133.42 | 94.59 | 76.63 | 80.25 | 65.43 |
| 58.40 | 162.77 | 89.62 | 69.12 | 90.74 | 52.74 |

Table 11. Individual data for liver cholesterol

| Control | Hyperlipidemia | Hyperlipidemia  + orlistat  (30 mg/kg b.w.) | Hyperlipidemia  +CGA7  (50 mg/kg b.w.) | Hyperlipidemia  +CGA7  (100 mg/kg b.w.) | Hyperlipidemia  +CGA7  (150 mg/kg b.w.) |
| --- | --- | --- | --- | --- | --- |
| 25.09 | 62.47 | 38.82 | 56.75 | 48.50 | 32.87 |
| 19.99 | 75.11 | 53.21 | 44.07 | 42.68 | 44.87 |
| 20.11 | 60.95 | 36.19 | 63.32 | 67.80 | 20.32 |
| 25.74 | 78.85 | 50.21 | 32.16 | 42.62 | 24.65 |
| 26.81 | 62.11 | 61.70 | 44.12 | 30.18 | 24.33 |
| 21.49 | 77.58 | 44.08 | 56.91 | 41.15 | 25.62 |
| 31.94 | 70.11 | 53.55 | 49.24 | 29.41 | 25.77 |
| 20.18 | 69.08 | 59.54 | 41.24 | 54.50 | 29.83 |

Table 12. Individual data for liver triglycerides

| Control | Hyperlipidemia | Hyperlipidemia  + orlistat  (30 mg/kg b.w.) | Hyperlipidemia  +CGA7  (50 mg/kg b.w.) | Hyperlipidemia  +CGA7  (100 mg/kg b.w.) | Hyperlipidemia  +CGA7  (150 mg/kg b.w.) |
| --- | --- | --- | --- | --- | --- |
| 53.25 | 86.37 | 49.76 | 68.64 | 59.68 | 50.50 |
| 42.94 | 73.62 | 77.97 | 59.84 | 60.24 | 53.57 |
| 53.93 | 98.66 | 70.95 | 65.23 | 64.23 | 24.48 |
| 55.26 | 94.97 | 68.65 | 63.25 | 60.21 | 27.62 |
| 46.52 | 80.50 | 68.16 | 36.42 | 55.25 | 26.28 |
| 41.02 | 97.09 | 51.68 | 50.26 | 50.68 | 48.46 |
| 57.26 | 76.31 | 68.53 | 46.84 | 75.70 | 31.47 |
| 53.93 | 95.90 | 61.45 | 70.93 | 53.25 | 25.97 |
